# Case Report: Endoscopic-guided electrocautery ablation of a vaginal hemorrhagic lesion in a dog

**DOI:** 10.3389/fvets.2026.1875395

**Published:** 2026-07-23

**Authors:** Sarah R. Michalak, Carrie A. Palm, Dennis J. Woerde, William T. N. Culp

**Affiliations:** 1 William R. Pritchard Veterinary Medical Teaching Hospital, University of California, Davis, Davis, CA, United States; 2 Department of Veterinary Medicine and Epidemiology, UC Davis Weill School of Veterinary Medicine, Davis, CA, United States; 3 Medipaws Veterinary Specialist Centre, Leichardt, NSW, Australia; 4 Department of Veterinary Surgical and Radiological Sciences, UC Davis Weill School of Veterinary Medicine, Davis, CA, United States

**Keywords:** electrocautery ablation, endoscopic-guided, minimally invasive, vaginal lesion, vulvar hemorrhage

## Abstract

A 7-year-old female spayed Labrador retriever was presented for a 3-year history of progressive hemorrhage from the vulva. The dog previously underwent cystourethroscopy and vaginoscopy 1 year prior, and a small, raised hemorrhagic lesion was identified in the caudal vaginal wall. Following pinch biopsies of the lesion, bleeding ceased during the remainder of the procedure but recommenced within a week following discharge from the hospital. Histopathology of the vaginal biopsy revealed chronic neutrophilic and lymphoplasmacytic vaginitis. One year later, the dog was reevaluated for persistent and progressive bleeding from the vulva. At that time, dual phase abdominal computed tomography identified a focal, 0.5 cm x 1.0 cm region of asymmetric contrast enhancement within the vaginal wall. Cystourethroscopy and vaginoscopy identified a progressively enlarged bleeding polypoid mass lesion in the caudodorsal vaginal wall. Biopsy of the lesion followed by monopolar electrocautery ablation of the proliferative tissue was performed. Histopathology revealed hemorrhage lined by a layer of stratified squamous epithelium and neutrophilic exocytosis with no neoplastic cells identified. The underlying etiology of the lesion was not definitively determined. Vulvovaginal bleeding resolved following the procedure and remains resolved at the time of writing 7 months later. This case report describes successful endoscopic-guided electrocautery ablation of a vaginal hemorrhagic lesion in a dog.

## Introduction

Vulvovaginal hemorrhage in the absence of estrus-related hormonal influence in dogs can be caused by diseases localized to the urogenital tract such as neoplasms, trauma, foreign bodies, inflammation, and vascular anomalies or by systemic disorders of coagulation (1–3). Vulvar and vaginal neoplasms are relatively rare in dogs (4), and most tumors are benign and of smooth muscle or fibrous tissue origin (1). In veterinary medicine, treatment of vaginal masses has included surgical excision, often combined with ovariohysterectomy in intact females (1, 5). Surgical excision of various bleeding vaginal lesions has been reported in dogs, including vaginal hemangiomas (6, 7), vulvovaginal hemangiosarcoma (8), and vaginal ectatic vessels (9).

Minimally invasive treatment of congenital anomalies of the vagina such as endoscopic-guided laser ablation of vestibulovaginal septal remnants are routinely performed in dogs (10). However, minimally invasive treatment of vaginal masses and other bleeding vaginal lesions has not been reported and could offer several advantages over open surgical techniques including reduced morbidity and faster recovery. This case report describes successful resolution of chronic vaginal hemorrhage in a dog using endoscopic-guided electrocautery ablation.

## Case description

A 7-year-old female spayed Labrador retriever was evaluated for a 3-year history of vulvovaginal hemorrhage without a history of pollakiuria, stranguria, or dysuria. Prior diagnostic investigation included multiple normal complete blood counts and a normal prothrombin time (PT) and activated partial thromboplastin time (aPTT). Although additional testing for primary hemostatic disorders was not performed, the dog had no other history of bleeding and had undergone routine ovariohysterectomy in the past without hemorrhagic complication. Biochemistry panels demonstrated persistent mild azotemia (creatinine 1.6 mg/dL; reference interval (RI) 0.8–1.5 mg/dL), consistent with the dog’s known diagnosis of IRIS stage II chronic kidney disease. Additionally, three incidences of *E. coli* bacteriuria were identified, two of which coincided with vulvar bleeding, though no other lower urinary tract signs were noted at the time bacteriuria was diagnosed. Antimicrobial therapy failed to resolve the vulvar hemorrhage, and negative urine cultures were obtained on multiple occasions with the vulvar bleeding present.

**Figure 1:**
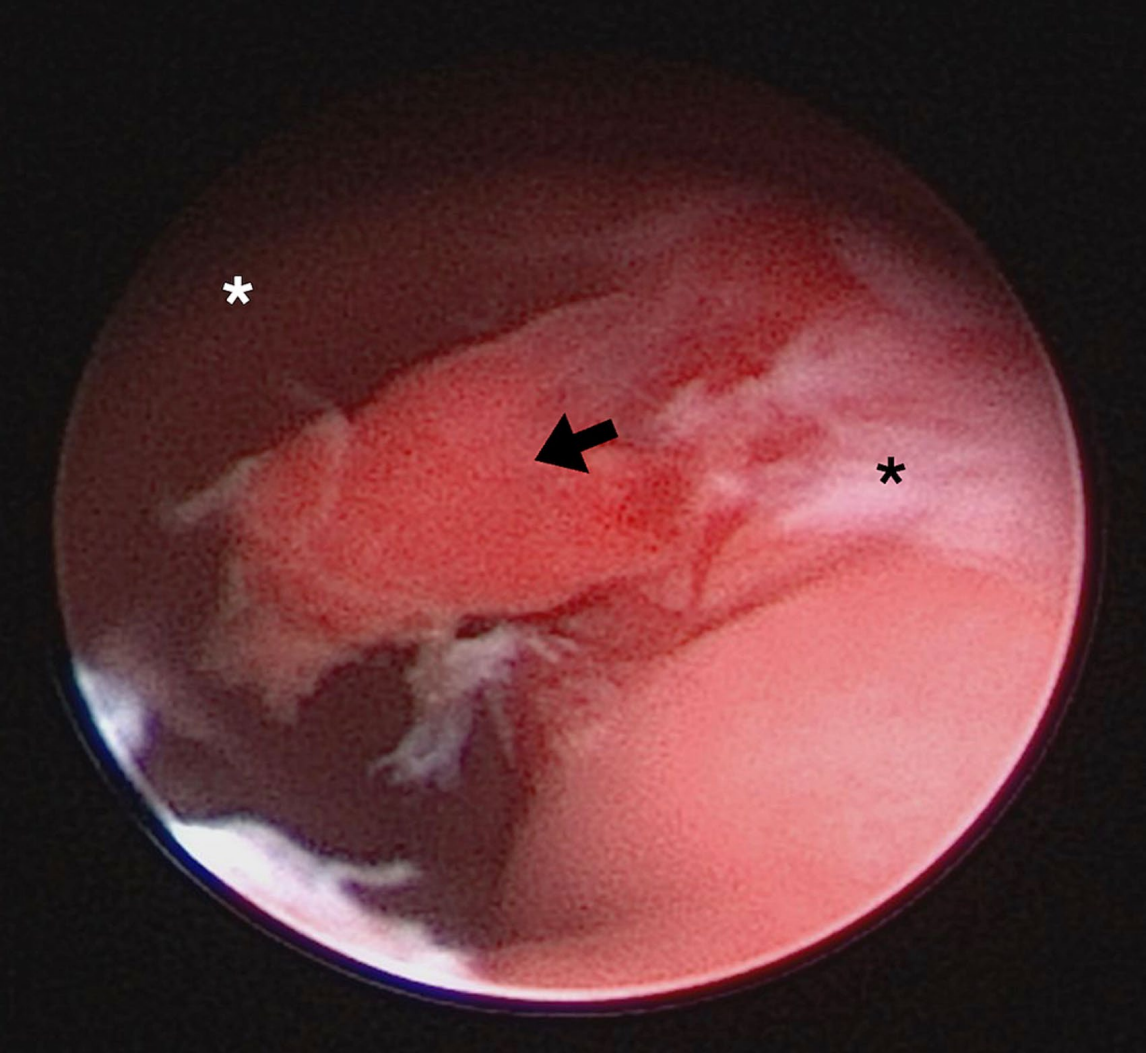
Endoscopic image from the patient’s first cystourethroscopy and vaginoscopy, identifying pale, irregular mucosa on the right side of the caudal vagina (black star) with adherent hyperemic tissue, suspected to represent a blood clot (black arrow), projecting into the vaginal lumen (white star).

Cystourethroscopy and vaginoscopy were initially performed 2 years after the development of bleeding. Pertinent findings included pale, irregular mucosa on the right caudal vaginal wall surrounded by a blood clot (Figure 1). No other vaginal, urethral, or bladder mucosal abnormalities were identified. Biopsies of the abnormal area within the vaginal mucosa were collected, and histopathology showed chronic neutrophilic and lymphoplasmacytic vaginitis. Bleeding resolved during the remainder of the procedure but restarted within a week following discharge. The dog’s vulvovaginal bleeding continued and gradually increased in frequency and severity, particularly when the dog was active or stressed. In the year between evaluations, the dog had undergone episioplasty with her primary care veterinarian to address her hooded vulva, under the assumption that recurrent bacteriuria could be related to abnormal vulvar conformation and a contributing factor to the bleeding. The patient recovered uneventfully from the procedure and multiple negative urine cultures were obtained in the recheck interim despite ongoing bleeding.

**Figure 2:**
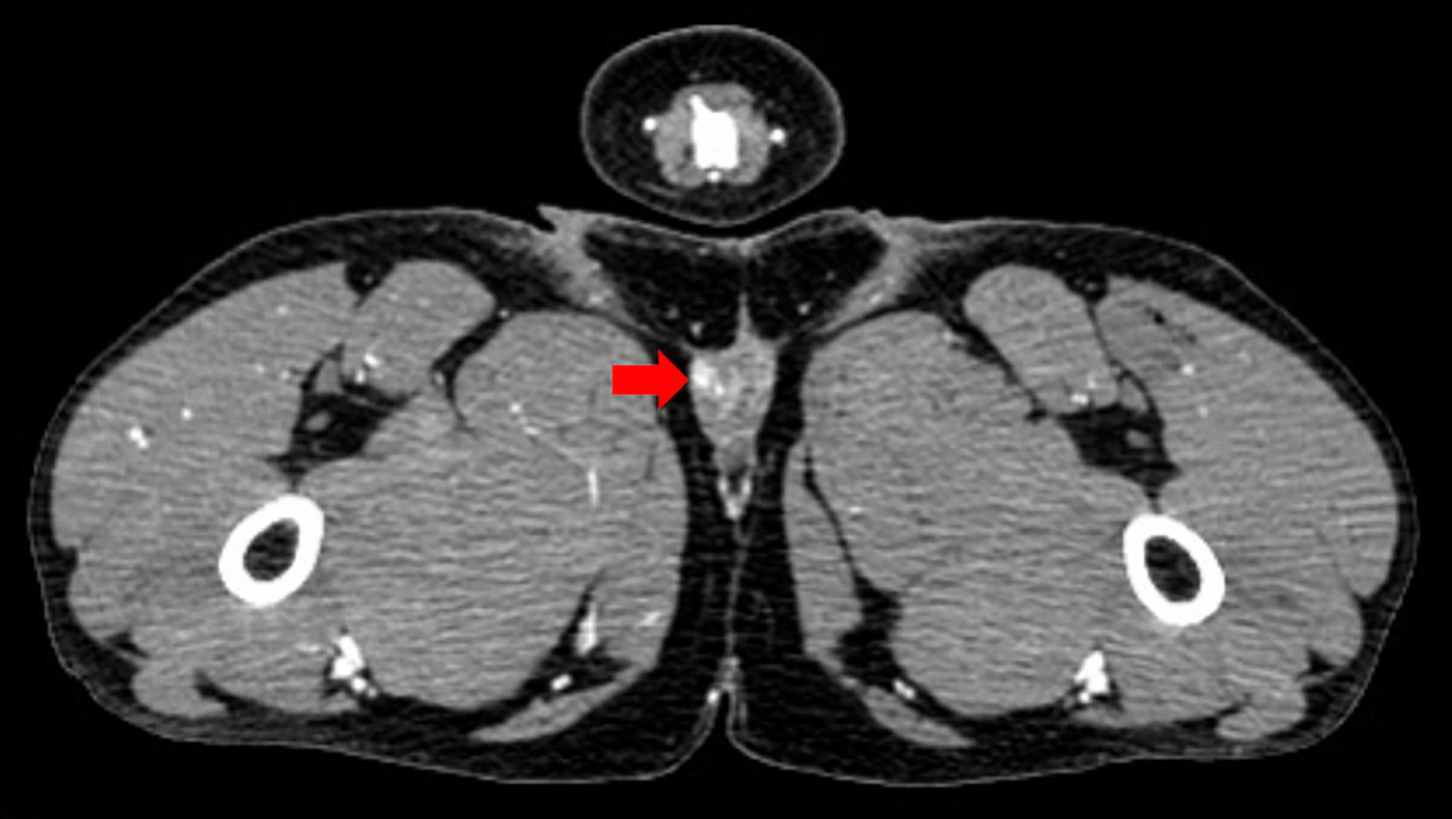
Arterial phase contrast-enhanced computed tomography scan, revealing a focal, 0.5 cm x 1.0 cm region of asymmetric contrast enhancement within the right dorsal aspect of the vaginal wall (red arrow).

By the time of the second presentation, the owner reported nearly constant bleeding from the vulva, consisting of small drops of blood. On physical examination, the frank hemorrhagic vulvar discharge was noted. A complete blood count and biochemistry panel were within normal limits aside from stable mild azotemia (creatinine 1.7 mg/dL; RI 0.8–1.5 mg/dL). Dual phase (arterial and venous) contrast-enhanced computed tomography (CT) images of the abdomen were acquired (GE Lightspeed 16, GE Medical Systems, Milwaukee, WI; Iohexol contrast media Omnipaque 350; GE Healthcare Inc.) with the patient under general anesthesia. The images were reconstructed in soft tissue and bone algorithms. The resulting images revealed a focal 0.5 cm x 1.0 cm region of asymmetric contrast enhancement within the right dorsal aspect of the vaginal wall (Figure 2). The uterine stump was unremarkable, and no abdominal or inguinal lymphadenopathy was seen. Focal inflammation, an ill-defined nodule, or a small vascular anomaly were considered differentials for the vaginal lesion based on imaging findings. Following the CT scan, the dog was referred for repeat cystourethroscopy and vaginoscopy with the goal of removing the lesion with either a minimally invasive approach or surgical intervention. An aerobic urine culture was negative one week prior to proceeding with endoscopy.

**Figure 3:**
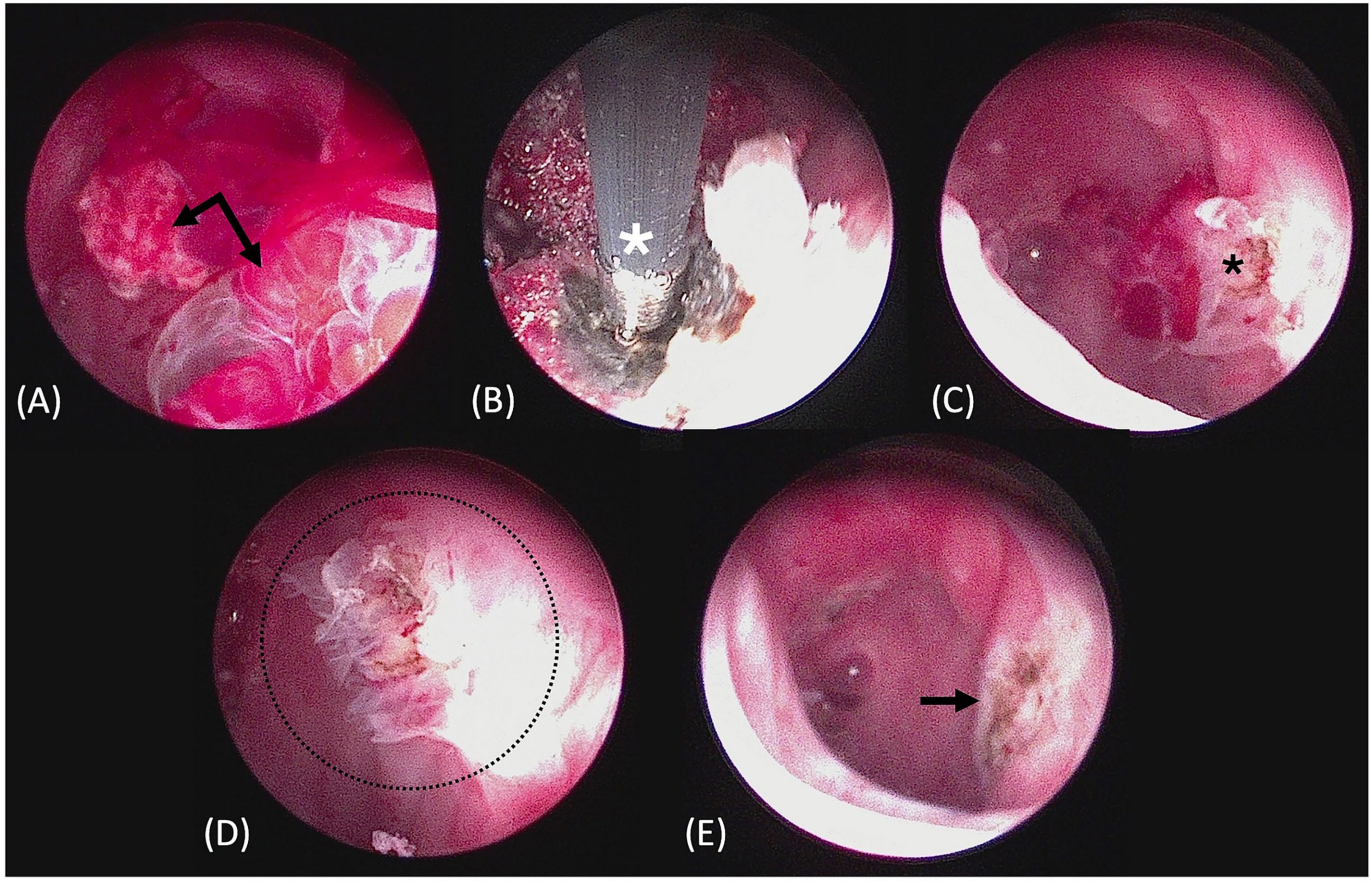
Endoscopic images from the patient’s second cystourethroscopy and vaginoscopy, revealing **(A)** a hemorrhagic multilobulated polypoid lesion (black arrows) at the right side of the caudal vagina, progressively enlarged compared to the prior endoscopy (performed approximately 1 year earlier, shown in Figure 1). **(B)** Monopolar coagulating ball electrode (white star) was used to apply electrocautery to the lesion first across the stalk (**C**; black star) and then across the base of the lesion (**D**; black dotted circle) to all grossly abnormal tissue, resulting in a smooth mucosal surface (**E**; black arrow).

Cystourethroscopy and vaginoscopy were performed under general anesthesia using a 14 Fr diameter 30° rigid cystoscope (Karl Storz SE & Co KG, Tuttlingen, Germany). The patient was placed in right lateral recumbency with the grounding pad placed securely on the patient’s right flank. Throughout the procedure, 5% dextrose in sterile water was used as an irrigation fluid to prevent anticipated hemorrhage from obscuring visualization and to serve as a nonconductive irrigation fluid for electrocautery. Upon entrance into the vestibule, mild hemorrhage was noted. Mild glomerulation-like lesions of the bladder mucosa were identified, but the remainder of the urethra and bladder appeared within normal limits. A hyperemic polypoid lesion was identified at the right dorsal aspect of the caudal vagina, presumed to be the source of the bleeding (Figure 3A). Several blood vessels supplying the proliferative tissue were visible at the base. On the contralateral vaginal wall, the mucosa contained multifocal pale/white plaques on the surface. No other masses or vascular lesions were visible in the vagina. A single biopsy of the bleeding vaginal lesion was performed with flexible biopsy forceps prior to introducing the electrocautery device. Minimal bleeding was induced. A 6 Fr open-ended ureteral catheter was then passed through the working channel of the endoscope. A 3 Fr, 110-centimeter length monopolar coagulating ball electrode (Karl Storz SE & Co KG, Tuttlingen, Germany) was passed through the ureteral catheter and advanced into the vaginal lumen. The electrode was interfaced with a monopolar electrosurgical unit (Conmed, Sabre Genesis, Largo, FL) operating in blend mode at a power setting of 7–15 W, applied to the base of the proliferative tissue. Electrocautery was directly applied to any visible blood vessels spanning across the base of the lesion. Flexible biopsy forceps were then passed again through the working channel of the scope and used to collect freed pieces of tissue and debulk any remaining pieces of the lesion. All samples were placed in formalin and submitted for histopathology. The base of the mass, which now appeared as mildly irregular mucosa, was cauterized at low power (5–7 Watts) across the surface using the electrode. During cauterization, no significant smoke accumulation or loss of endoscopic visualization occurred. To minimize the risk of thermal spread and collateral tissue injury, electrocautery was applied using the lowest effective power setting in short, controlled bursts with continuous endoscopic visualization. Care was taken to avoid prolonged activation at a single site, reducing the risk of vaginal wall perforation. No significant bleeding requiring additional intervention was encountered (Figure 3).

The region of contralateral mucosa containing superficial plaques was also sampled for culture; however, submittable pieces of tissue could not be obtained as the plaques were minimally adhered to the mucosa and were mechanically dislodged by the biopsy forceps. Bupivacaine (2 mg/kg) diluted 1:1 with sterile 0.9% saline was instilled into the vagina through the scope and allowed to dwell. Total procedure time was approximately 1 h, with active electrocautery applied intermittently for an estimated cumulative duration of 15 min.

The patient recovered uneventfully from anesthesia and was managed post-procedurally with intravenous fluids and a single dose of methadone 0.2 mg/kg intravenously. No vulvar hemorrhage was noted in hospital following the procedure. The patient was deemed suitable for discharge the following day and sent home with carprofen 1.5 mg/kg by mouth every 12 h for 3 days. The owner provided an update about 10 days following discharge and reported return to normal activity and appetite with no signs of vulvar hemorrhage. Additionally, no lower urinary tract signs nor overt signs of discomfort had been noted by the owner during her recovery.

**Figure 4:**
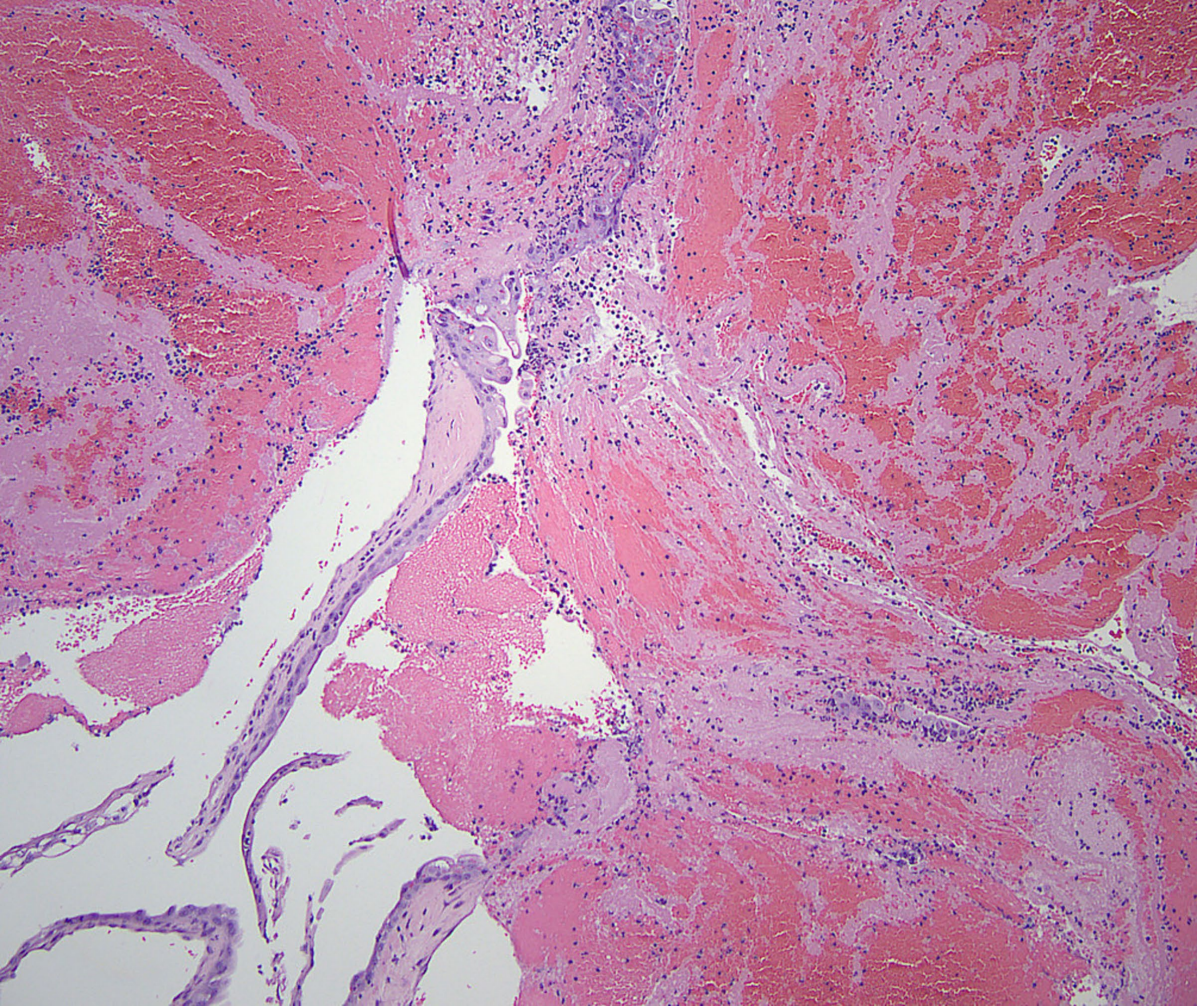
Histopathologic evaluation (H&E stain) of a representative section taken from endoscopic biopsies of a hemorrhagic vaginal lesion, revealing sheets of free erythrocytes lined by a layer of stratified squamous epithelium. No neoplastic cells were identified.

Four formalin-fixed tissue samples from the mucosal lesion were submitted for histopathology, consisting of three 1–2 mm fragments and one 1 cm tissue sample. Histopathology revealed lakes of free erythrocytes, fibrin, and fewer neutrophils with some sections containing pieces of sloughed stratified squamous epithelium with a small layer of underlying collagenous connective tissue. Mild anisocytosis and dysplastic change of the epithelial cells were appreciated. No evidence of neoplasia or deeper inflammatory infiltrate was evident in the examined sections, and the pathogenesis of the vaginal lesion could not be determined by histopathologic evaluation. Cautery artifact was not noted to impact the interpretation of the sections (Figure 4).

**Figure 5:**
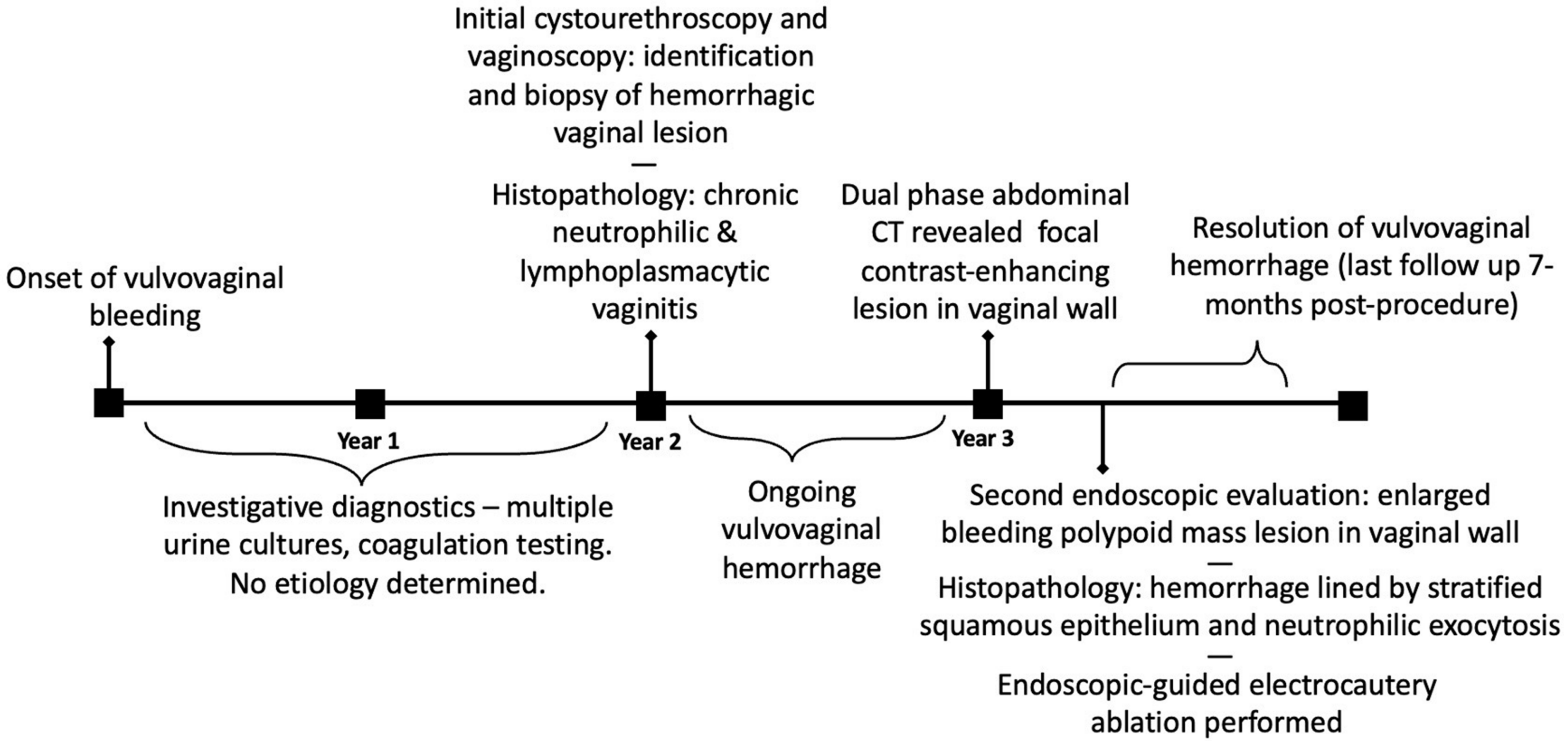
Timeline reflecting the clinical course of the patient in this report.

During a phone consultation with the owner at 7 months post-procedure, no recurrence of vulvovaginal bleeding was reported and no lower urinary tract signs were noted in the interim. The timeline of the patient’s clinical course is summarized in Figure 5.

## Discussion

To the author’s knowledge, this is the first known report of endoscopic-guided electrocautery ablation applied to a bleeding vaginal lesion in a dog. Traditional treatment for vaginal masses in dogs involves surgical excision, for which approaches by laparotomy and episiotomy have been described (1). Total vaginectomy was pursued for a dog with vaginal hemorrhage caused by vaginal vascular ectasia in one case report, which successfully resolved vulvar bleeding for 19 months following surgery after which time bleeding recurred (9).

Although multiple studies have reported favorable prognoses following surgical resection of vaginal tumors (1, 5), the application of minimally-invasive approaches to conditions historically addressed with surgical intervention is of growing interest in veterinary medicine. For example, endoscopic mucosal resection and snare electrocautery have been applied to colorectal masses in dogs (11, 12) and duodenal and gastric polyps in cats (12, 13). Endoscopic-guided electrocautery ablation has also been reported as an effective treatment for a tracheal liposarcoma in a dog (14). A multitude of endoscopic-guided ablation techniques in the urogenital tract of dogs have been described including cystoscopically-guided laser ablation of hemorrhagic lesions in the bladder and urethra (15), palliative diode laser ablation of transitional cell carcinomas (16), and laser ablation of vestibulovaginal septal remnants (10), intramural ectopic ureters (17), and ureteroceles (18). Minimally invasive approaches to vaginal bleeding using electrosurgery are commonly employed in human medicine, including endoscopic polypectomy for the management of female reproductive polyps (19) and techniques of endometrial ablation to address abnormal uterine bleeding (20).

A minimally invasive technique using endoscopic-guided electrocautery was elected for the dog in this report based on the relatively small and focal appearance of the vaginal mass and the suspicion of benign etiology based on initial histopathology results and the prolonged history of bleeding. The owner was also reluctant to pursue more invasive approaches due to the perception that the bleeding was not impacting the dog’s overall health or quality of life. The procedure was performed without complication and was well-tolerated by the dog in terms of post-procedural comfort and rapid return to normal behavior and activity. No further vulvovaginal hemorrhage nor lower urinary tract signs have been noted through the time of writing at 7 months post-procedure, even during exercise and stress, though repeat vaginoscopy has not been performed due to lack of clinical need.

Several limitations should be considered when making conclusions about this report. As a single-patient case report, the safety, efficacy, indications, lesion recurrence risk, and generalizability of endoscopic-guided electrocautery for treatment of hemorrhagic vaginal lesions cannot be established. While no complications were observed in this case, potential risks of monopolar electrocautery within the vagina include thermal injury to non-target tissue, perforation, pain, vaginal stenosis/stricture when treating luminal lesions that extend circumferentially, and incomplete lesion ablation, which should be discussed in depth prior to applying this treatment modality to clinical cases. Follow-up was limited to owner telephone consultation at 7 months after treatment, without repeat vaginoscopy, computed tomography, or histopathologic confirmation of complete lesion resolution. Although no recurrence of clinical signs was reported, 7 months remains a relatively short follow-up period for a lesion of uncertain etiology.

A major limitation of the present report is that the definitive etiology of the bleeding vaginal lesion remains undetermined. Histopathologic evaluation of the biopsy specimens revealed hemorrhage lined by stratified squamous epithelium without features sufficient to definitively diagnose a hemangioma, vascular ectasia, inflammatory polyp, or neoplastic process. The collected biopsy specimens represented mucosal tissue only and did not contain deeper vaginal wall layers; therefore, it is not possible to definitively rule out the presence of submucosal or muscular vascular abnormalities or neoplasia. It is also possible that the initial biopsy procedure performed the year prior to ablation altered the appearance of the lesion or contributed to subsequent lesion enlargement by inducing local hemorrhage or inflammation.

The etiology and clinical significance of the pale mucosal plaques located contralateral to the primary lesion also remain unknown because attempts to obtain diagnostic samples for histopathology and microbial culture were unsuccessful. Consequently, it remains unclear whether the plaques represented reactive inflammatory change due to chronic irritation from contact with the proliferative lesion or instead represent encrusting or infectious mucosal lesions that promoted chronic inflammation of the vagina and subsequent tissue proliferation. Based on the unremarkable appearance of the mucosa surrounding the plaques, a benign etiology is prioritized. Although no progression or associated clinical abnormalities were noted by the owner during follow-up, the lack of cytologic or histopathologic evaluation of these plaques represents a limitation of this report.

In the present case, the authors consider a localized vascular abnormality, such as vascular ectasia, a small vascular malformation, or a superficial hemangioma, to be the most likely explanation for the chronic hemorrhage. However, these diagnoses were not confirmed. A vascular inflammatory polyp is also considered as a possible etiology. A stimulus for chronic inflammation was not identified, though idiopathic vaginitis, a prior vaginal foreign body, or exuberant inflammation associated with recurrent bacterial vaginitis are considered, and an inflammatory etiology could relate to the uncharacterized mucosal plaques. A neoplastic process, including hemangiosarcoma or other vascular tumor, are considered much less likely given the long clinical course and lack of malignant features or evidence of metastatic disease.

The lack of a definitive diagnosis for the hemorrhagic vaginal lesion further limits conclusions regarding the broader applicability of endoscopic-guided electrocautery for the treatment of bleeding vestibulovaginal lesions of differing etiologies in dogs. Nevertheless, the successful resolution of clinical signs and absence of reported recurrence during follow-up suggest that endoscopic-guided electrocautery may represent a viable minimally invasive therapeutic option for select cases of focal vaginal hemorrhage.

In conclusion, endoscopic-guided electrocautery ablation was an effective and well-tolerated minimally invasive treatment of a hemorrhagic vaginal lesion in this dog. This report underscores the importance of considering minimally invasive options for veterinary patients and employing established electrosurgical techniques for new applications. Further investigation is warranted to better define the indications, safety, and long-term outcomes of this technique in dogs with hemorrhagic vaginal lesions.

## Data Availability

The original contributions presented in the study are included in the article/supplementary material, further inquiries can be directed to the corresponding author/s.
